# Gut Microbiome Alterations in Patients With Visceral Obesity Based on Quantitative Computed Tomography

**DOI:** 10.3389/fcimb.2021.823262

**Published:** 2022-01-20

**Authors:** Hang Yan, Qian Qin, Jengfeng Chen, Su Yan, Tiantian Li, Xinxin Gao, Yang Yang, Ang Li, Suying Ding

**Affiliations:** ^1^ Health Management Center, The First Affiliated Hospital of Zhengzhou University, Zhengzhou, China; ^2^ College of Public Health, Zhengzhou University, Zhengzhou, China; ^3^ Gene Hospital of Henan Province, The First Affiliated Hospital of Zhengzhou University, Zhengzhou, China

**Keywords:** visceral obesity, gut microbiota, metagenomics, metabolic pathways, quantitative computed tomography

## Abstract

The gut microbiota is crucial in the pathogenesis of obesity. Abdominal obesity is known to significantly increase the risk of metabolic syndrome and cardiovascular disease, so further study is needed to investigate the changes of intestinal microorganisms in patients with excessive visceral fat. In our study, 41 people (n = 41) with normal body mass index (BMI) (18.5 ≤ BMI < 23.9) were included and divided into the low visceral fat area (L-VFA) group (n = 23, VFA < 100 cm^2^) and the high visceral fat area (H-VFA) group (n = 18, VFA ≥ 100 cm^2^). Several clinical indicators of the H-VFA group were significantly higher than those of the L-VFA group, including the waist circumference (WC), the fasting blood glucose (FBG), the triglyceride (TG), the total cholesterol (TC), the low-density lipoprotein cholesterol (LDL), the serum uric acid (SUA), the white blood cell count (WBC), the blood neutrophil count (NEC), and the blood lymphocyte count (LYC). Using whole-genome shotgun sequencing, we found that the types of the intestinal microbiota of H-VFA patients were different from those of the L-VFA patients, with 18 bacteria enriched in the H-VFA group and nine bacteria in the L-VFA group. A total of 16 species of gut microbes showed a strong correlation with VFA, and *Escherichia coli* has the strongest correlation, followed by *Mitsuokella unclassified*, *Bifidobacterium longum*, *Escherichia unclassified*, *Ruminococcus torques*, *Dialister succinatiphilus*, *Eubacterium hallii*, and *Ruminococcus gnavus*. Compared to the VFA, only two species show a strong correlation with BMI and WC. Further functional genetic studies suggested that the degradation of short-chain fatty acids (SCFAs) and the generation of lipopolysaccharide (LPS) might be related to visceral fat accumulation. Together, visceral fat was more closely correlated with the gut microbiome compared with BMI and WC. It suggested an intrinsic connection between the gut microbiome and visceral fat and its related metabolic disorders. Specific microbial species and pathways associated with visceral fat accumulation might contribute to new targeted therapies for visceral fat and its metabolic disorders.

## 1 Introduction

Previous studies have found that compared to peripheral obesity, visceral obesity is a more significant risk factor for cardiovascular and cerebrovascular diseases, diabetes, and hypertension (Ibrahim, 2010). However, inappropriate lifestyles have led to more cases of “invisible obesity” (a normal BMI but excessive visceral fat area), aggravated the earlier onset of chronic diseases, and brought severe economic and living burdens to families and society (Kelly et al., 2008). As we all know, diet control, exercise, medication, and surgical interventions can be used to control body weight. However, they may induce joint damage, side effects of drugs, and surgical trauma. Therefore, are there other methods that can be used to improve weight?

In the last decade, the development of high-throughput platforms has allowed researchers to concurrently determine the composition and functions of the gut microbiota in diseases (Yadav and Chauhan, 2021). Accumulating evidence has demonstrated the close relationship between obesity and microorganisms (Marchesi et al., 2016); probiotics may be able to become a new way to improve obesity. Recently, studies have shown that obesity reduces the diversity of intestinal microbiota composition (Guo et al., 2018). Compared to children of normal BMI, the ratio of Firmicutes to Bacteroides was higher in obese children diagnosed with exceeding BMI (Riva et al., 2017). The relative proportion decreased along with weight loss (Ley et al., 2006). However, several recent studies have confirmed that VFA is more related to the intestinal microbiome than BMI and waist circumference (WC) (Beaumont et al., 2016; Ozato et al., 2019; Nie et al., 2020). A cross-sectional study in Japan found that *Blautia* was significantly negatively correlated with the VFA measured by a visceral fat meter but did not correlate with the BMI (Ozato et al., 2019). Furthermore, using the DXA method to measure the VFA, Beaumont et al. found that the VFA and *Oscillospira* members had the strongest associations measured by 16sRNA in the Twins UK cohort (Beaumont et al., 2016). Furthermore, a study observed that probiotics supplement can reduce VFA (Pedret et al., 2019), such as the liver fat area in mice (Munukka et al., 2017). Studies have shown that the metabolites of gut microbes may explain this phenomenon: the correlation between obesity and gut microbes (Aoun et al., 2020). On the one hand, LPS produced by intestinal microbes will increase intestinal permeability to induce chronic low-grade inflammation, and it causes insulin resistance by activating TLR4 (Cani et al., 2007). On the other hand, the SCFAs (McNabney and Henagan, 2017) (such as butyric acid) can reduce the systemic inflammatory response by maintaining the intestinal epithelial barrier. It has also been found that SCFA supplementation can reduce insulin resistance by using animal models (Gao et al., 2009).

Although the relationship between gut microbiota and obesity as assessed by BMI has been proven, it is necessary further to analyze the relationship between gut microbiota and visceral fat. Therefore, the subjects with normal BMI but excessive visceral fat in this study were included and diagnosed with a metagenomic to study the relationship between intestinal microbiota and “invisible obesity”.

## 2 Materials and Methods

### 2.1 Study Population

A total of 41 people measured with VFA by QCT and metagenomic sequencing of fecal were included. Their QCT measurement, fecal collection, and biochemical index testing were performed on the same day. All 41 subjects with a normal BMI (18.5 ≤ BMI < 23.9) were divided into the control group (low visceral fat area, L-VFA) (VFA < 100 cm^2^) with an average age of 48.78 ± 4.45 years and the high visceral fat area group (H-VFA) (VFA ≥ 100 cm^2^) (Irlbeck et al., 2010) with an average age of 50.89 ± 4.47 years. All studies involving human participants were reviewed and approved by the ethics committee from the First Affiliated Hospital of Zhengzhou University (approval number: 2018-KY-56). Exclusion criteria were as follows: (1) patients with metabolic diseases such as lipoatrophy, hyperthyroidism, hypothyroidism; (2) patients with major malignant diseases (tumors, etc.); (3) those who had taken cholesterol-lowering drugs, antihypertensive drugs, or oral hypoglycemic drugs; patients with hypertension, impaired glucose regulation, and diabetes; and (4) patients with incomplete laboratory tests results.

### 2.2 Basic Information and Biochemical Indicators

Basic information of the subjects, including age, previous medical history, and medication history, was collected. The WC, weight, and height (computerized body scale, Sonka, SK-X80, Shenzhen) were measured, and the BMI (kg/m^2^) was calculated as weight/height^2^. The blood pressure (OMRON medical automatic electronic blood pressure monitor, HBP-9021, Dalian) was measured simultaneously. Fasting venous blood (8–10 h) was extracted overnight, and its serum was separated within 2 h. The following indexes were calculated using a Roche automatic biochemical analyzer Cobas 8000 (Roche, Mannheim, Germany) on the same day: white blood cell count (WBC), blood neutrophil count (NEC), blood lymphocyte count (LYC), fasting blood glucose (FBG), glycated hemoglobin (HbA1C), low-density lipoprotein cholesterol (LDL), high-density lipoprotein cholesterol (HDL), triglyceride (TG), total cholesterol (TC), and serum uric acid (SUA).

### 2.3 Measurement of the VFA

Philips Brilliance ICT Elite FHD CT was used for spiral scanning. CT followed the set low-dose spiral scanning conditions. Scanning method: the tube voltage was 120 kV, and the tube current adopted automatic tube current technology. Volunteers were divided into two groups according to their body mass: ① body mass >70 kg, Dose Right Index 10, and reference tube current 41 mAs; ② body mass ≤70 kg, Dose Right Index 3, and reference tube current 19 mAs. Reconstruction method: all models are reconstructed by iterative reconstruction technology (Iterative Model Reconstruction, IM R. IeveI is 2). Pitch is 0.984, scanning layer thickness and layer spacing are both 5 mm, recombination layer thickness and layer spacing are both 1 mm, x-ray tube rotation speed is 0.5 S/r, matrix is 512 s/r spacing, display field (display field of view, DFoV) is 500 mm, and bed height is based on the mid-axillary line as the scanning baseline. The subjects were placed in a supine position with hands on top of their heads, and images were scanned at end-expiratory breath-holding. Data were uploaded to the Mindways QCT PRO V6.1 workstation to calculate the VFA (Cheng et al., 2018).

### 2.4 DNA Extraction, Shotgun Metagenomic Sequencing, and Quantity Control of Reads

DNA was extracted from a total of 41 stool samples using the MagPure Stool DNA KF kits, according to the manufacturer’s instructions. The DNA extraction kit was provided by Angen Biotech Co., Ltd. (Yayingshi Road, Luogang District, Guangzhou, China). We used a unique stool collection tube (Sarstedt stool collection tube, Germany). The sample collection volume is about 1 mg. After the stool is collected, it is immediately cooled and stored at -20°. 0.2 mg of frozen feces was taken for DNA extraction. The DNA library construction based upon the DNA nanospheres (DNB) and the shotgun metagenomic sequencing based upon the combined probe anchoring synthesis (CPAs) were carried out for all samples (MGI2000, MGI, Shenzhen, China). The all-inclusive accuracy (OA ≥ 0.8) control strategy detailed above was used to perform quality control (QC) on the original reads to filter out the low-quality reads (Fang et al., 2018).

### 2.5 Microbiome Composition and Function Profiling

Standard relative abundance values of all species at all levels were obtained by MetaPhlAn2, which was used to carry out the metagenomic classification of sequenced libraries. Primarily, it contrasted between the sequence and marker. The MetaPhlAn2 (Truong et al., 2015) classifier lastly distinguished metagenomic reads against the precomputed marker catalog using nucleotide BLAST searches to provide abundances in clade for either one or more sequenced metagenomes. Subsequently, the total number of reads in each clade was normalized by the classifier through the nucleotide length of its markers. The relative abundance of each taxonomic unit was calculated, considering any titles specific to subclades. Therefore, the microbial clade anomaly was estimated by normalizing read-based counts by the average genome size of each clade. Using these methods, we generated a map of the gut microbes containing bacteria, archaea, eukaryotes, and viruses. Community functional profiles of gut microorganisms were further created using HumanN2 (HMP Unified Metabolic Analysis Network 2) (Fang et al., 2018; Franzosa et al., 2018; Li et al., 2021).

### 2.6 Statistical Analysis

Statistical analysis was performed using the R procedure (version 4.0.2), and standardized statistical methods were used to analyze demographic information and laboratory test results. Continuous variables were expressed as mean ± standard deviation, while classified variables were expressed as counts. For the difference between groups, normality and homogeneity of variance were first tested, and p ≥0.05 was considered as normality and homogeneity of variance. Then, the parametric test (t-test) or non-parametric test (rank-sum test) was used for statistical analysis, and the chi-square test was used for determining the differences in constituent ratios between groups. The Shannon, Obs, Hellinger, JSD, Bray, and Pearson indexes of each sample were calculated by using the “vegan” package in R. R program “ade4” was used to perform principal coordinate analysis (PCoA) for visual analysis. The Spearman correlation was used to analyze the correlation between clinical characteristics and microbiome or metabolic pathways. STAMP (version 2.1.3) was used to analyze the differences in the microbiota at the phylum levels, species levels, and pathways. White’s non-parametric test was applied to calculate the differences between groups, with Benjamini–Hochberg FDR for multiple test correction. Corrected p < 0.05 was considered statistically significant. The species with low occurrences (positivity rates <10%) were dismissed before analyzing the differential microbiotas.

## 3 Results

### 3.1 Clinical Characteristics of Subjects

A total of 41 subjects were included, including 23 subjects in the L-VFA group (mean age 48.78 ± 4.45 years) and 18 subjects in the H-VFA group (mean age 50.89 ± 4.47 years). There were no significant differences in the BMI, age, systolic blood pressure (SBP), diastolic blood pressure (DBP), HbA1C, or HDL between the two groups. The levels of the WC, FBG, TG, TC, LDL, SUA, WBC, NEC, and LYC in the H-VFA group were significantly higher than those in the L-VFA group (*p <*0. 05) (**Table 1**).

**Table 1:** The essential characteristics and laboratory test results.

	H-VFA (n = 18)	L-VFA (n = 23)	*p*
Age	50.89 ± 4.47	48.78 ± 4.45	0.141
Gender	male6; female12	male5; female18	0.406
BMI	22.23 ± 1.18	22.01 ± 1.04	0.541
WC	79.47 ± 4.65	74.08 ± 3.68	0.001*
VFA	150.75 ± 37.05	75.35 ± 15.72	<0.001*
SBP	121.89 ± 13.05	115.57 ± 19.21	0.239
DBP	77.11 ± 10.76	72.43 ± 12.79	0.221
FBG	5.53 ± 0.68	4.93 ± 0.32	0.010*
HbA1C	5.81 ± 0.49	5.58 ± 0.37	0.098
TC	5.30 ± 1.23	4.50 ± 0.84	0.020*
TG	1.67 ± 0.78	1.06 ± 0.44	0.006*
LDL	3.32 ± 1.02	2.64 ± 0.79	0.023*
HDL	1.47 ± 0.40	1.66 ± 0.37	0.123
SUA	311.28 ± 76.56	262.41 ± 53.37	0.023*
WBC	6.51 ± 1.26	5.19 ± 1.14	0.001*
NEC	3.80 ± 0.94	2.97 ± 0.87	0.006*
LYC	2.16 ± 0.50	1.74 ± 0.42	0.007*

WC, waist circumference; BMI, body mass index; VFA, visceral fat area; SBP, systolic blood pressure; DBP, diastolic blood pressure; FBG, fasting blood glucose; HbA1c, glycated hemoglobin A1c; TC, total cholesterol; TG, triglyceride; HDL, high-density lipoprotein; LDL, low-density lipoprotein; SUA, serum uric acid; WBC, white blood cell; NEC, neutrophil count; LYC, lymphocyte count. Student’s t-tests were used to compare the differences between the H-VFA (n = 18) and the L-VFA (n = 23) groups. *p < 0.05.

### 3.2 Analysis of Microbiota Diversity

**Figure 1:**
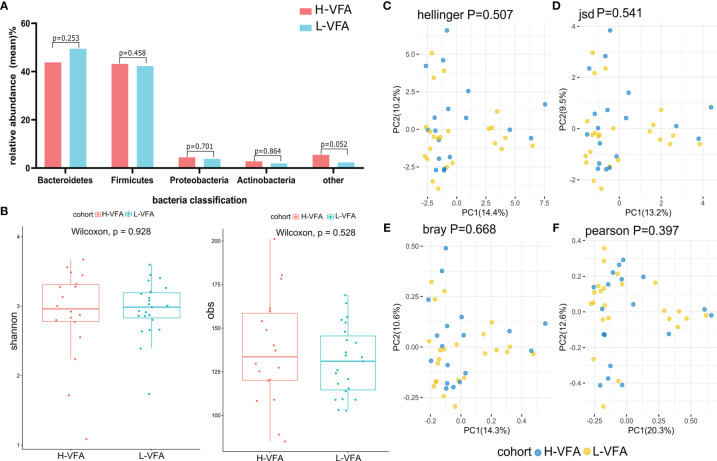
Microbiome composition and diversity. **(A)** No significant differences were found in the phyla of the H-VFA group and L-VFA group (*p* > 0.05). **(B)** Both Shannon index and obs index measured alpha diversity in comparisons between the H-VFA group and L-VFA group. There was no significant difference in the level of microbial species between the two groups (p = 0.928 and p = 0.528). **(C–F)** Beta diversity between the H-VFA group and L-VFA group. Beta diversity was calculated based on the Hellinger distance, JSD distance, Bray distance, or Pearson distance. There were no significant differences between the H-VFA group and the L-VFA group.

The classification was sought through the basis of the identified sequences into phyla and genera based on their closest matches in the database referred. Generally, gut microbiota was dominated by four abundant phyla that were present in the H-VFA group and the L-VFA group at very similar levels: Proteobacteria (4.41% and 3.77%), Bacteroidetes (43.78% and 49.43%), Actinobacteria (2.78% and 1.95%), and Firmicutes (43.17% and 42.23%). There were no significant differences between the H-VFA and L-VFA groups (p > 0.05) (**Figure 1A**).

At the species level, no significant differences between the microbiomes of the H-VFA and the L-VFA subjects were observed in alpha diversity, which was measured by Shannon index and Obs index (p = 0.928 and p = 0.528) (**Figure 1B**). To compare the species-level beta diversity of the H-VFA and L-VFA groups, the Hellinger distance, Jensen–Shannon divergence (JSD) distance, Bray distance, and Spearmen distance were calculated. Results showed no significant differences in the species-level beta diversity between the H-VFA and L-VFA groups (p = 0.507, p = 0.541, p = 0.668, and p = 0.397, respectively, **Figures 1C–F** **)**.

### 3.3 Analysis of the Microbiota Composition and Associations Between the Gut Microbiome and Clinical Characteristics

#### 3.3.1 Analysis of the Microbiota Composition

**Figure 2:**
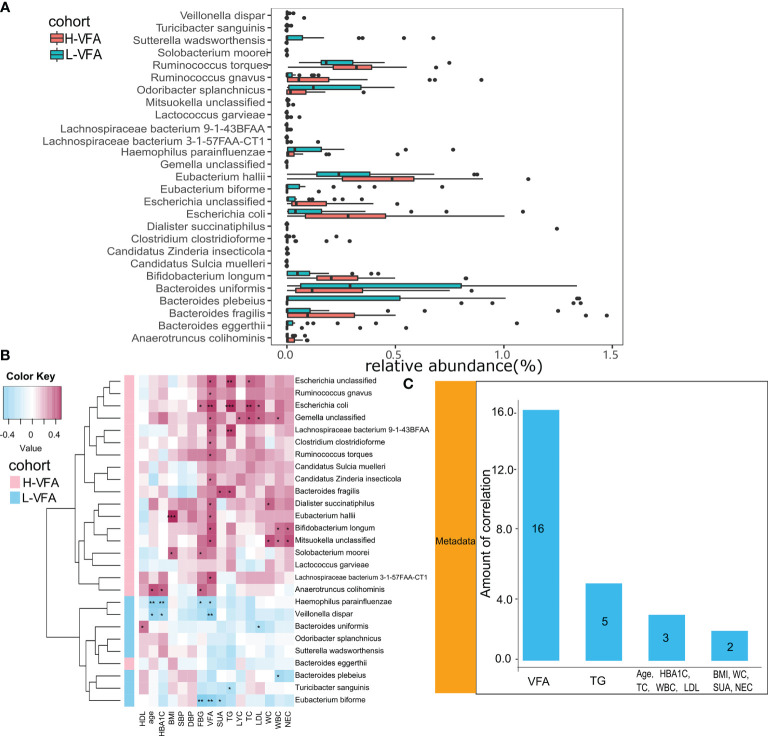
**(A)** The relative abundance of bacterial species between the H-VFA group and the L-VFA group. Wilcoxon test analysis of the relative abundance of bacterial species showed significant differences in 27 species, with nine species enriched in the L-VFA group and 18 enriched in the H-VFA group. **(B**) Correlations between bacteria and clinical characteristics. Pink cells denote positive correlations, whereas blue cells depict negative correlations. *, **, and *** denote p < 0.05, p < 0.01, and p < 0.001, respectively. **(C)** The number of significant correlations for the eleven risk factors.

The species-level analysis found that 44 species in the H-VFA group were significantly different from the L-VFA group (White’s non-parametric test and Benjamini–Hochberg FDR, p < 0.05). There were significant differences in the abundance of 27 species. After excluding the species with low incidence and abundance (p < 0.05; **Figure 2A**), nine were enriched in the L-VFA group, and 18 were increased in H-VFA group. The Wilcoxon test showed that the L-VFA group supplemented the following bacteria: *Eubacterium biforme*, *Bacteroides (Bacteroides eggerthii*, *Bacteroides uniformis*, and *Bacteroides plebeius*), *Odoribacter splanchnicus*, *Sutterella wadsworthensis*, *Turicibacter sanguinis*, *Haemophilus parainfluenzae*, and *Veillonella dispar*, whereas we found that *Ruminococcus (Ruminococcus gnavus, Ruminococcus torques), Lachnospiraceae (Lachnospiraceae bacterium 3-1-57FAA-CT1* and *Lachnospiraceae bacterium 9-1-43BFAA), Candidatus (Candidatus sulcia muelleri* and *Candidatus zinderia insecticola)*, and *Escherichia (Escherichia coli* and *Escherichia unclassified)* were enriched in the H-VFA group. At the same time, *Anaerotruncus colihominis*, *Bacteroides fragilis*, *Bifidobacterium longum*, *Clostridium clostridioforme*, *Eubacterium hallii*, *Gemella unclassified*, *Lactococcus garvieae*, *Mitsuokella unclassified*, *Dialister succinatiphilus*, and *Solobacterium moorei* were also concentrated in the H-VFA group (**Figure 2A**).

#### 3.3.2 Associations Between the Gut Microbiome and Clinical Characteristics

Spearman’s correlation analysis analyzed the correlations between species abundances and clinical characteristics. Thirteen species enriched in the H-VFA group were positively correlated with the VFA, with *Escherichia coli* having the strongest correlation (r = 0.444, p < 0.05), followed by *Mitsuokella unclassified* (r = 0.392, p < 0.05), *Bifidobacterium longum* (r=0.389, p < 0.05), and *Escherichia unclassified* (r = 0.384, p < 0.05). Additionally, among the 11 risk factors, VFA had the largest number of correlations with bacterial species (n = 16, p < 0.05), followed by TG (n = 5, p < 0.05), HbA1c (n =3, p < 0.05), TC (n = 3, p < 0.05), and LDL (n = 3, p <0.05) (**Figure 2C**). Only two species (*Eubacterium hallii* and *Solobacterium moorei*) were positively associated with the BMI, and two species (*Dialister succinatiphilus* and *Mitsuokella unclassified*) were positively correlated with the WC (**Figure 2C**). Species related to the VFA were positively correlated with metabolic indicators (FBG, TG, TC, LDL, and SUA) and inflammation indicators (WBC, NEC, and LYC) in the H-VFA group, such as *Escherichia unclassified*, *Escherichia coli*, *Gemella unclassified*, and *Lachnospiraceae bacterium 9143BFAA*, whereas *Veillonella dispar*, *Haemophilus parainfluenzae*, and *Eubacterium biforme* enriched in the L-VFA group showed a stronger negative correlation with the VFA than with the BMI or the WC. Species related to the VFA were negatively correlated with metabolic indicators and inflammatory indicators in the L-VFA group (**Figure 2B**).

### 3.4 Functional Shifts in the Microbiome Characteristics of Different Subjects and Associations Between Functional Shifts and Clinical Characteristics

#### 3.4.1 Functional Shifts in the Microbiome Characteristics of Different Subjects

**Figure 3:**
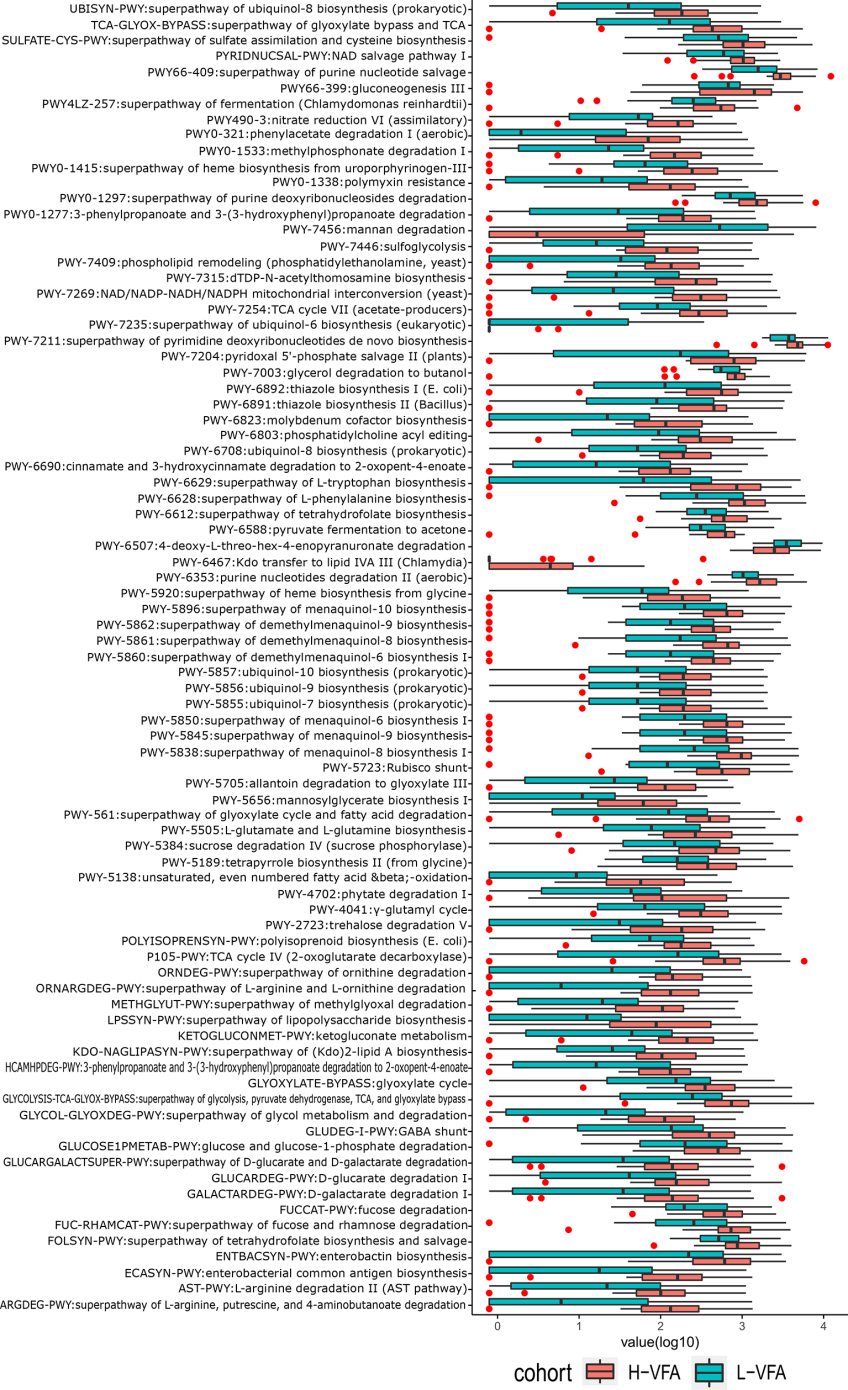
The functional shifts of bacterial species between the H-VFA group and the L-VFA group. Eighty-three pathways were significantly different between the two groups, and 80 enriched the H-VFA subjects. Within the 80 H-VFA enriched pathways, 24 ways were responsible for the degradation of short-chain fatty acids, production of lipopolysaccharides, and metabolism of carbohydrates, fatty acids, lipids, and amino acids.

We also constructed functional profiles for each sample using 89 microbial MetaCyc pathways. After removing low abundance pathways, a total of 83 MetaCyc pathways were found to be significantly different between the H-VFA group and the L-VFA group (**Figure 3**), 80 of which were enriched in the H-VFA subjects. Among the 80 pathways, four were involved in the production of LPS, namely, ECASYN-PWY, PWY0-1338, KDO-NAGLIPASYN-PWY, and PWY-6467. PWY-5088 was involved in the degradation of SCFAs; six pathways were responsible for generating or degrading carbohydrates, including PWY-2723, PWY66-399, FUCCAT-PWY PWY-5384, GGLUCOSE1PMETAB-PWY, and PWY-7315. PWY-5138, LPSSYN-PWY, PWY-7409, PWY-561, and PWY-6803 were responsible for generating or degrading fatty acids and lipids. Eight pathways accountable for the production or degradation of amino acids were PWY-5505, AST-PWY, PWY-6628, 3-HYDROXYPHENYLACETATE-DEGRADATION-PWY, ARGDEG-PWY, GLUDEG-I-PWY, PWY-6629, and ORNARG DEG-PWY. Other pathways were responsible for electron transport, carboxylate degradation, degradation of aromatic compounds, and so on. Three pathways were enriched in the L-VFA group, with PWY-7456 involved in the degradation of carbohydrates, PWY-7235 involved in electron transport, and PWY-6507 involved in the degradation of sugar derivatives (**Figure 3**).

#### 3.4.2 Associations Between Functional Shifts in the Microbiome Characteristics and Clinical Characteristics

**Figure 4:**
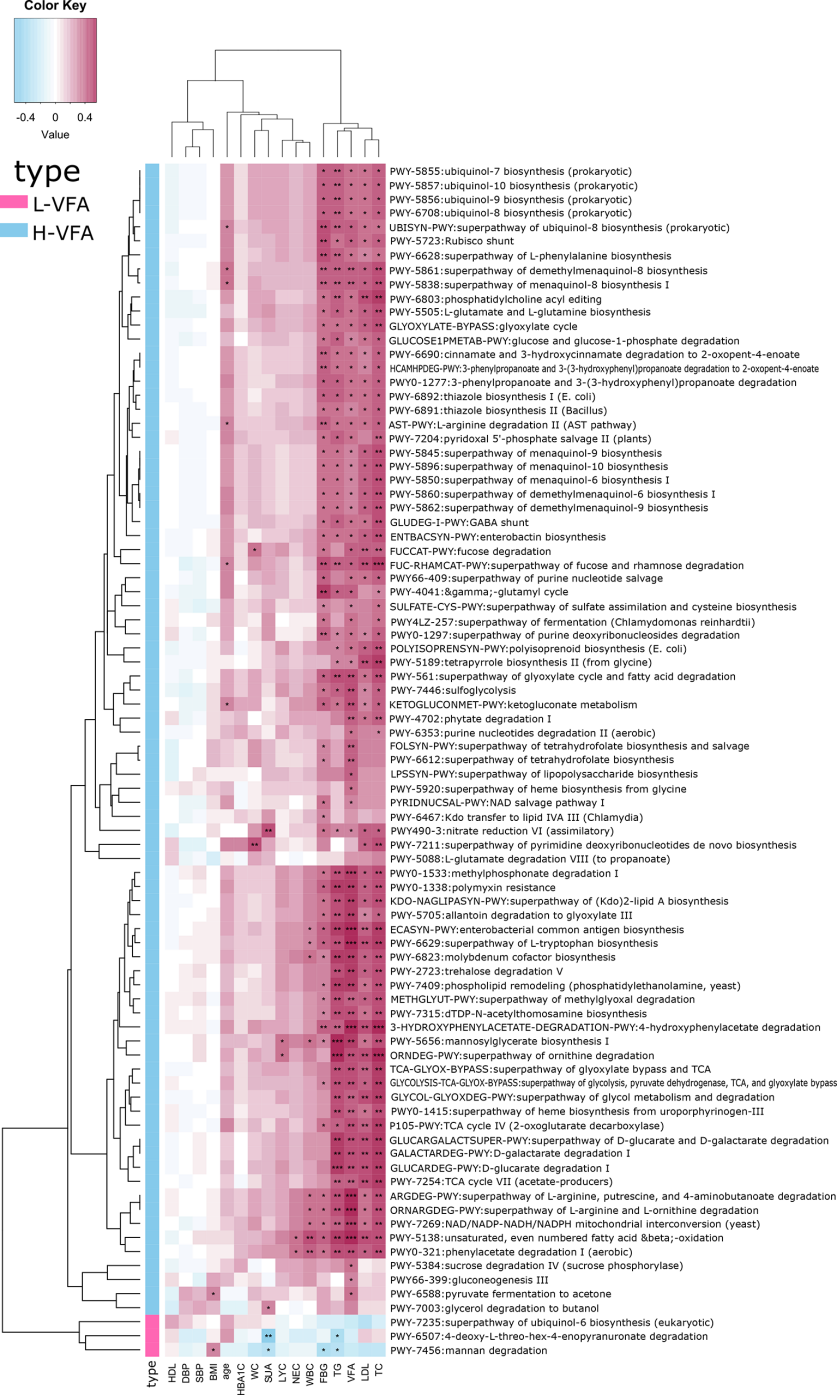
Association between functional shifts in the microbiome characteristics and clinical characteristics. Cell color indicates the correlation type (pink: positive, blue: negative). *, **, and *** denote p < 0.05, p < 0.01, and p < 0.001, respectively. In the H-VFA group, almost all the pathways were positively correlated with the VFA. Only one pathway (PWY-6588) was positively correlated with the BMI, and two pathways (FUCCAT-PWY, PWY-7211) were positively correlated with the WC.

Spearman’s correlation analysis analyzed the correlations between functional shifts and clinical characteristics. In the H-VFA group, almost all the pathways were positively correlated with VFA, TG, TC, LDL, FBG, SUA, WBC, NEC, and LYC, while negatively correlated with the HDL; only one pathway (PWY-6588) was positively correlated with the BMI, and two pathways (FUCCAT-PWY, PWY-7211) were positively correlated with the WC. In the L-VFA group, three pathways were negatively correlated with the FBG, SUA, TG, and inflammation indicators (WBC, NEC, and LYC), while positively correlated with the HDL (**Figure 4**).

## 4 Discussion

More and more studies have demonstrated that the risk of obesity-related metabolic diseases and cardiovascular diseases is closely related to fat distribution, especially visceral fat. BMI as the traditional anthropometric parameter can only evaluate the total body fat, but not the fat distribution. In recent years, it has been discovered that central obesity with excessive accumulation of visceral fat significantly increases the risk of atherosclerosis, type 2 diabetes, cancer, and all-cause mortality (Liu et al., 2019; Qiu et al., 2020; Xiong et al., 2021). This may be because the visceral fat is more active in storing dietary fatty acids, secreting adiponectin and inflammatory factors such as interleukin-6, tumor necrosis factor (TNF)-α, and colony-stimulating factor-1 (Fontana et al., 2007; Harman-Boehm et al., 2007; Cartier et al., 2008; Kovacova et al., 2012; Wang et al., 2012; Huang et al., 2015; Tsujimoto and Kajio, 2017; Barberio et al., 2019). Studies have shown that the metabolites of gut microbes in obese people may be involved in the inflammatory response. Although the relationship between intestinal microbiome and obesity (as assessed by the BMI) has been proven, analyzing the relationship between the intestinal microbiome and VFA may help to explore the potential “probiotics” of the “invisible obesity” people. It is the first time in China to use the QCT as the standard for diagnosing VFA to study the relationship between excessive visceral fat and the intestinal microbiome.

Our metagenomic study found that Odoribacter splanchnicus, Bacteroides (Bacteroides eggerthii, Bacteroides plebeius, and Bacteroides uniformis), Sutterella wadsworthensis, Haemophilus parainfluenzae, and Veillonella dispar were enriched in the L-VFA group. Bacteroides (Bacteroides plebeius and Bacteroides uniformis) may represent potential probiotics due to their enrichment in the L-VFA group relative to H-VFA subjects. A previous study investigated that Bacteroides uniformis protected against metabolic disorders and obesity in preclinical trials (Gomez Del Pulgar et al., 2020) and showed a stronger negative correlation with the VFA than the BMI or WC (Nie et al., 2020). This strain reduced body weight gain and improved lipid metabolism, diminishing liver steatosis and serum cholesterol and triglyceride levels in obese mice. It also reduced leptin levels and improved glucose metabolism since it decreased the fasting concentrations of serum glucose and insulin, therefore improving glucose tolerance. We found that Bacteroides uniformis were negatively correlated with LDL in our study. Apart from this, Bacteroides plebeius enriched in the L-VFA group was negatively correlated with WBC. A survey of intestinal microbes in children found that Bacteroides plebeius was enriched in normal weight (diagnosed by BMI). It correlated negatively with phenylalanine serum levels, a metabolite also found to be associated with obesity in children. In addition, Bacteroides plebeius can potentially be used for type 2 diabetes mellitus (T2DM) treatment (Wang et al., 2020), considering it supplying the human body with energy from dietary polysaccharides through active carbohydrate enzymes, or CAZymes (Hehemann et al., 2010). In our study, we found Bacteroides plebeius to be significantly increased in the L-VFA group.

Furthermore, our study showed that 13 species enriched in the H-VFA group were positively correlated with the VFA. *Escherichia coli* showed the strongest correlation, followed by *Mitsuokella unclassified, Bifidobacterium longum, Escherichia unclassified, Ruminococcus torques, Dialister succinatiphilus, Eubacterium hallii*, and *Ruminococcus gnavus*, whereas only two were positively correlated with the BMI and the WC, indicating a stronger correlation between VFA and gut microbiome than BMI and WC. *Ruminococcus gnavus* was enriched in obese rats (Petriz et al., 2014), but after a 6-month weight loss, it was decreased (Jie et al., 2021). This is consistent with our findings: *Ruminococcus gnavus* was enriched in the H-VFA group. Further studies found that *Ruminococcus gnavus* was negatively correlated with fat mass and leptin levels while not associated with the changes in food intake. Another study (Jie et al., 2021) suggested that the content of *Escherichia coli* was higher in obese preschool children. These studies found that the following reasons may cause this phenomenon: On the one hand, the reduction of short-chain fatty acids reduces the ability of the mucosal layer to exclude unfavorable bacterial strains. The abundance of metabolites of intestinal microorganisms reduces mucin production and affects the balance of the mucus layer of the gastrointestinal barrier to increased intestinal permeability; on the other hand, inflammatory polysaccharides produce inflammatory cytokines such as TNF-α through dendritic cells, which further leads to the destruction of the intestinal barrier function, causing or aggravating the occurrence of inflammation. *Escherichia coli* and *Ruminococcus gnavus* were enriched in the H-VFA group and were positively correlated with metabolic indicators, the WBC, and the NEC. Additionally, studies showed that *Dialister succinatiphilus* was increased in obese subjects and could be used as a biomarker for predicting obesity (Zhang et al., 2021). This is consistent with our results that *Dialister succinatiphilus* was increased in the H-VFA group. Previous studies also showed that *Dialister succinatiphilus* induced or aggravated host inflammatory response and insulin resistance by releasing more lipopolysaccharides. In our research, we also found that *Dialister succinatiphilus* was positively correlated with the WBC, the NEC, and the LYC.

To verify the above speculation, we conducted a functional genetic analysis and found that both the increased production of lipopolysaccharides and the decreased production of SCFAs occurred in the H-VFA group, which further indicated the associated mechanisms between visceral fat accumulation and gut microbes. The ECASYN-PWY, PWY0-1338, KDO-NAGLIPASYN-PWY, and PWY-6467 pathways were enriched in the H-VFA group; they were involved in the production of the lipopolysaccharide and positively correlated with the WBC and the NEC. Previous studies (Zhang et al., 2021) mentioned that lipopolysaccharide remains at low concentrations in healthy people but may reach high concentrations in obese individuals. It leads to metabolic endotoxemia. The level of lipopolysaccharide is related to a high-fat diet, an increase of which will cause local inflammation in the intestine, making the intestinal barrier weak and “leaky.” It can further lead to lipopolysaccharide absorption and interaction with CD14 located on immune cells (such as monocytes, macrophages, and intestinal epithelial cells). Receptor binding further triggers a systemic pro-inflammatory cascade and increases insulin resistance/obesity. When immune cells are activated for immune responses, the release of cytokines can weaken the insulin signal and cause insulin resistance over time (Gomes et al., 2018). Long-period systemic inflammation may progress into a state of chronic inflammation, which compromises the host’s glucose homeostasis and increases caloric intake, leading to weight gain.

We also found that PWY-5088 was enhanced in the H-VFA group, which could increase short-chain fatty acid degradation, leading to a decrease in the levels of SCFAs in the H-VFA group. Enrichment of PWY-5088 also positively correlated with metabolic indicators and the VFA but negatively correlated with the WBC and the NEC. The reduction of short-chain fatty acids in obese patients reduces the ability of the mucosal layer to exclude unfavorable bacterial strains. These bacteria or their metabolites can penetrate the intestinal barrier and invade surrounding tissues, stimulating the innate immune response and leading to chronic inflammation. Research showed that short-chain fatty acids upregulate anti-inflammatory cytokines and downregulate pro-inflammatory cytokines through different mechanisms, promote the homeostasis of the intestinal mucosa, and thus have a global anti-inflammatory effect (Morrison and Preston, 2016). In summary, we speculate that the intestinal microbiome in the H-VFA group increases the production of lipopolysaccharides and the degradation of SCFAs. They resulted in chronic low-grade inflammation and insulin resistance and led to metabolic disorders. This is consistent with the pathophysiological mechanism of metabolic disorders caused by excessive visceral fat. In addition, many critical metabolic pathways were modeled to be more active in the L-VFA group, such as the degradation of carbohydrates (PWY-7456) and sugar derivatives (PWY-6507). We found that the FBG of the L-VFA group was significantly lower than that of the H-VFA group, and PWY-7456 was negatively correlated with FBG in our study.

There are certainly some limitations. On the one hand, we did not collect the diet and nutritional intake; on the other hand, the inflammatory factors and metabolomics of the subjects were not measured to further reveal the correlation between visceral obesity and the intestinal microbiome. Future multiple omics studies and/or animal microbiota mechanism studies are needed to fully elaborate the relationship between the gut microbiota and visceral fat.

## 5 Conclusions

We established a metagenomic association between the gut microbiota and visceral fat accumulation and showed microbiota changes even in subjects with normal BMI and high VFA in this study. VFA had the strongest correlation with the species compared with other obese parameters (BMI and WC). The degradation of short-chain fatty acids and the production of lipopolysaccharides are closely related to visceral fat accumulation. *Bacteroides* (*Bacteroides plebeius* and *Bacteroides uniformis*) may represent potential probiotics to reduce the increase of visceral fat. Specific microbial species and pathways closely associated with visceral fat accumulation might lead to new therapies for metabolic disorders.

## Data Availability Statement

All shotgun whole genome sequences have been uploaded into the European Nucleotide Archive PRJEB36271. Demographic features and serum index used in this study are available only for academic usage. Please contact Professor SD (fccdingsy@zzu.edu.cn) for a metadata request.

## Ethics Statement

The studies involving human participants were reviewed and approved by the First Affiliated Hospital of Zhengzhou University (approval number: 2018-KY-56). The patients/participants provided their written informed consent to participate in this study.

## Author Contributions

HY, QQ, and JC wrote and edited the manuscript, and they contributed equally to this study. SY performed the data analysis. TL, XG, and YY helped to collect samples and data. SD and AL were in charge of the project. All authors contributed to the article and approved the submitted version.

## Funding

The study was supported by the National Natural Science Foundation of China (72101236), Henan Province Key Scientific Research Projects of Universities (21A320035), Youth Talent Promotion Project of Henan Province (2021HYTP052), Henan Province Medical Science and Technology Research Plan (LHGJ20200279), and Chinese National Science and Technology Major Project (2018ZX10305410).

## Conflict of Interest

The authors declare that the research was conducted in the absence of any commercial or financial relationships that could be construed as a potential conflict of interest.

## Publisher’s Note

All claims expressed in this article are solely those of the authors and do not necessarily represent those of their affiliated organizations, or those of the publisher, the editors and the reviewers. Any product that may be evaluated in this article, or claim that may be made by its manufacturer, is not guaranteed or endorsed by the publisher.
